# Supplementary material for: A new phylogenetic framework for the genus Kalanchoe (Crassulaceae) and implications for infrageneric classification
Source: Ann Bot. 2025 Jan 19;135(7):1311–28. doi: 10.1093/aob/mcaf004 (PMC12358026; doi:10.1093/aob/mcaf004)
Supplement: mcaf004_suppl_Supplementary_Figures_S1-S4 [file mcaf004_suppl_supplementary_figures_s1-s4.docx]

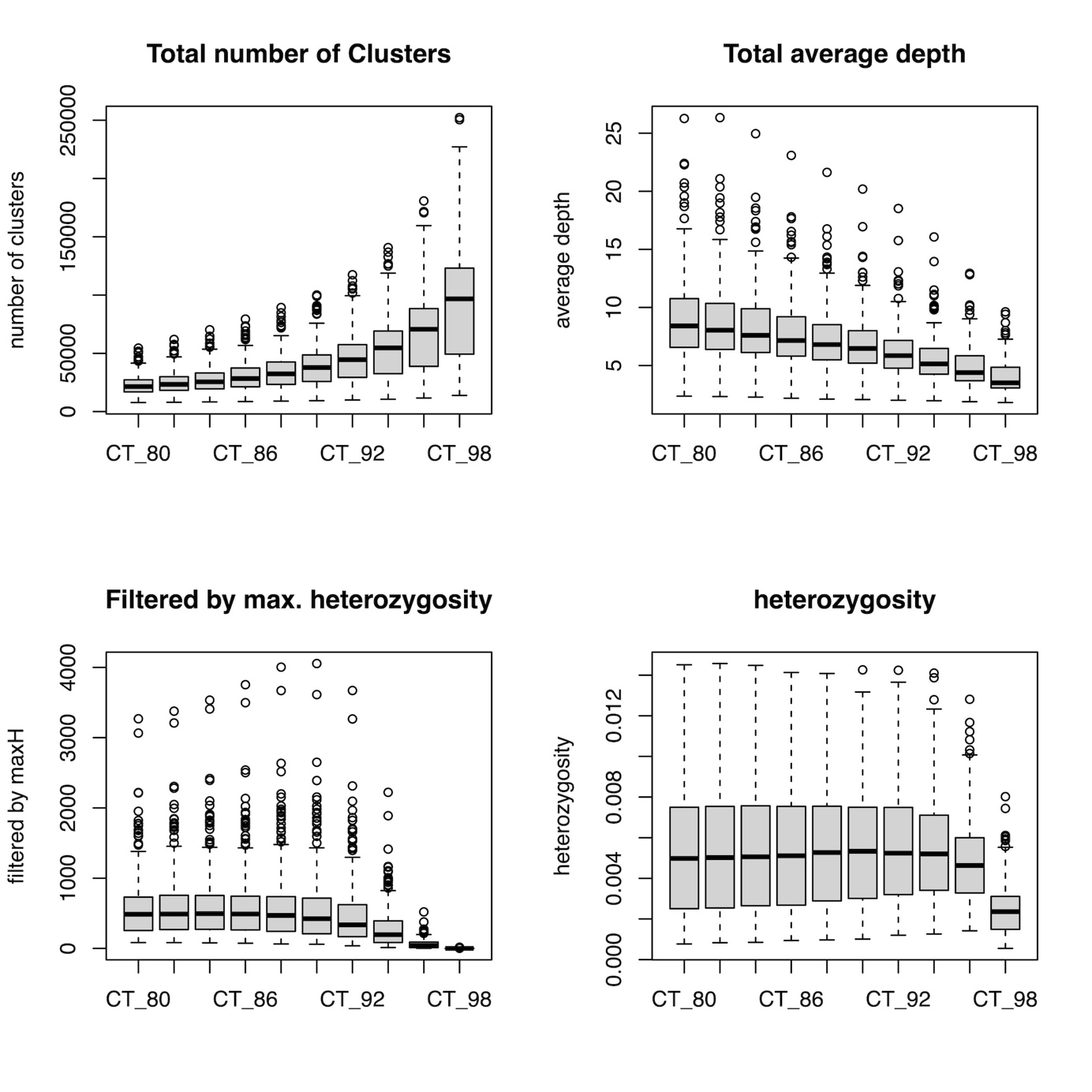


**Supplementary Figure S1a**. Comparison of within-sample clustering thresholds for four metrics that were evaluated for the assembly with the ipyrad pipeline. The total number of clusters, total average depth, loci filtered by maximum heterozygosity, and heterozygosity per sample are shown across within-sample clustering thresholds of 80–98% similarity between reads.


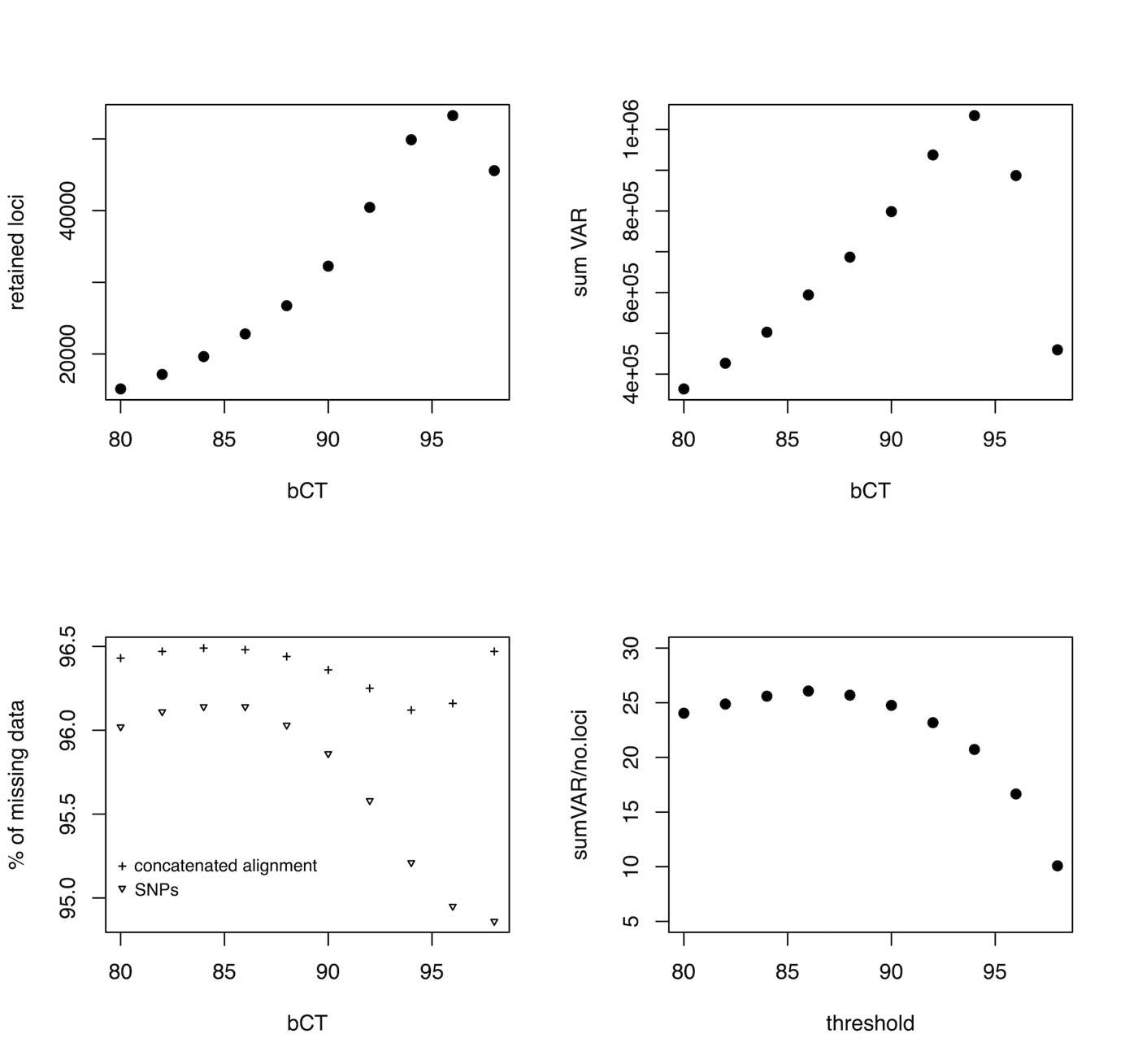


**Supplementary Figure S1b**. Comparison of between-sample clustering thresholds for four metrics that were evaluated for the assembly with the ipyrad pipeline. The total number of retained loci, the total number of variable sites, the percentage of missing data, and the average number of variable sites per locus are compared across between-sample clustering thresholds of 80–98% similarity between within-sample consensus sequence clusters.


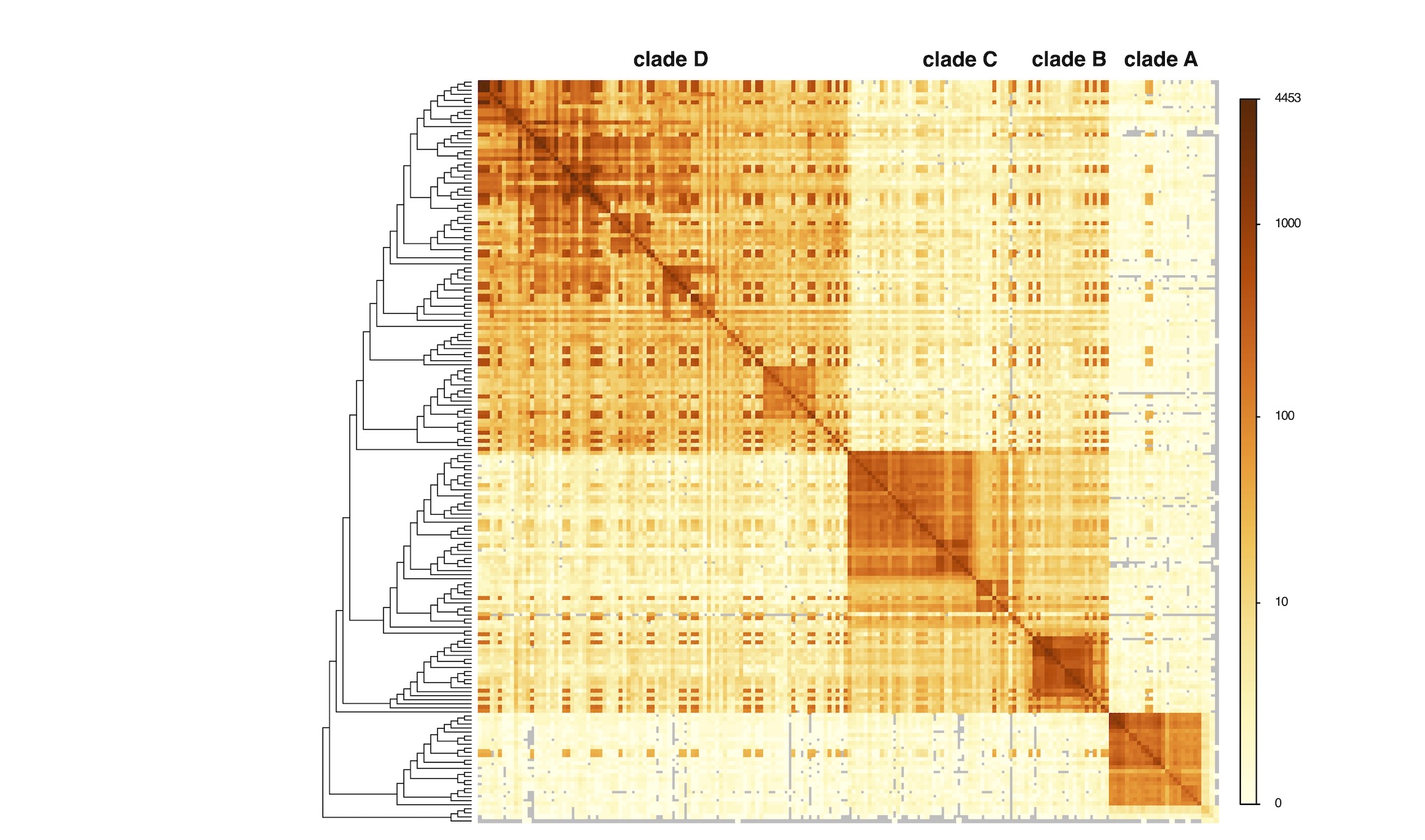


**Supplementary Figure S2**. Number of ddRADseq loci shared between samples. Rows and columns represent individual samples. They are ordered according to their placement in the concatenated tree, shown on the left. The diagonal entries show the number of loci recovered for each sample, and off-diagonal entries represent the number of shared loci between the samples. The colour coding is log-scaled with darker colours representing more shared loci between samples.


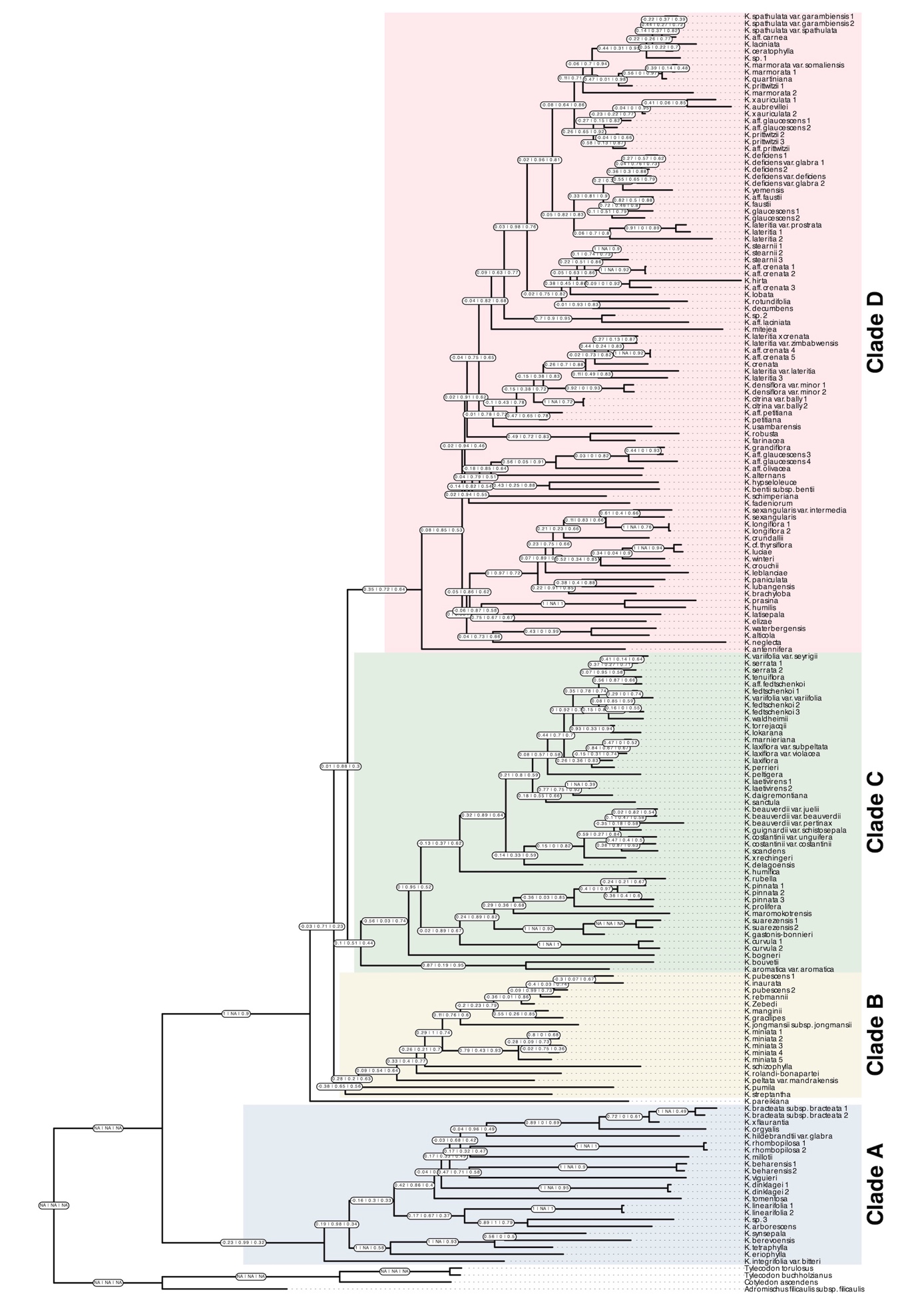


**Supplementary Figure S3**. Quartet sampling scores on maximum likelihood phylogeny of *Kalanchoe* *s.l.* inferred from the concatenated alignment of ddRADseq loci (concatenated tree). Quartet sampling scores (Quartet concordance (QC), quartet discordance (QD) and quartet informativeness (QI)) are shown for each branch separated by a vertical line.


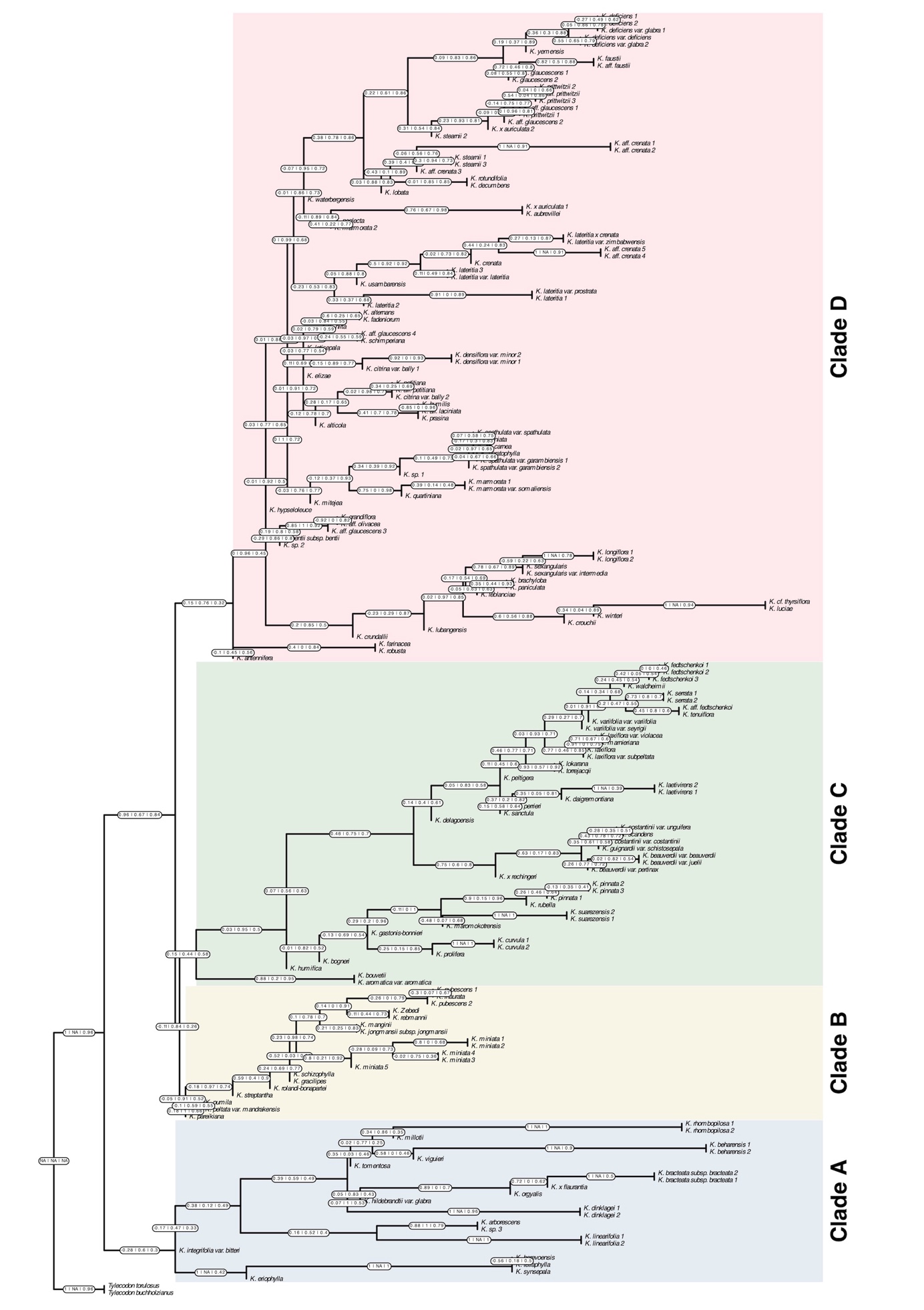


**Supplementary Figure S4a**. Quartet sampling scores on ddRADseq phylogeny of *Kalanchoe s.l.* inferred with a two-step coalescent approach (coalescent tree). Quartet sampling scores (Quartet concordance (QC), quartet discordance (QD) and quartet informativeness (QI)) are shown for each branch separated by a vertical line.


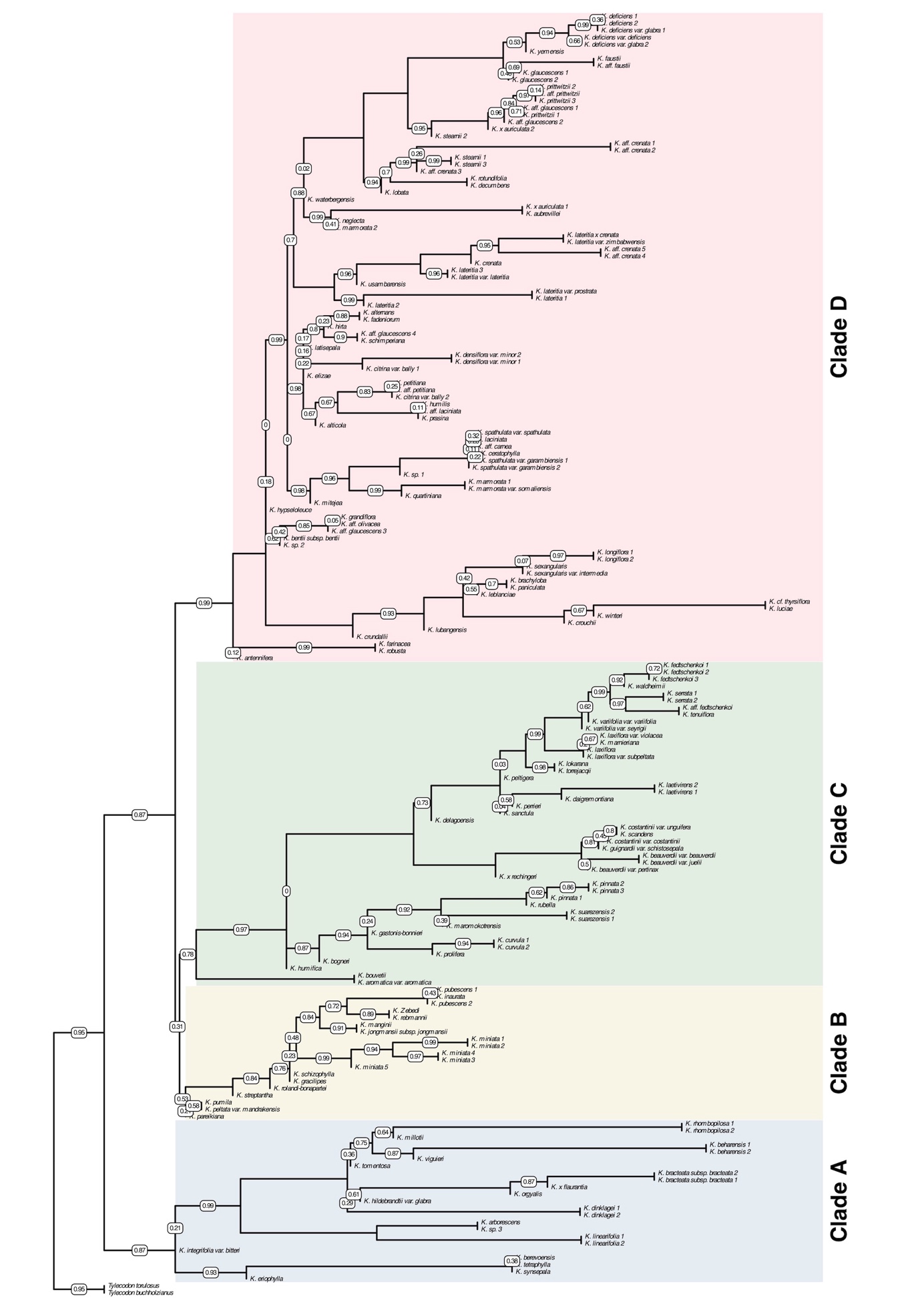


**Supplementary Figure S4b**. Local posterior probability support values on ddRADseq phylogeny of *Kalanchoe s.l.* inferred with a two-step coalescent approach (coalescent tree). local posterior probability support values are shown only for nodes that do not receive maximum support.
